# Conservation agriculture enhances soil and water conservation and crop yield in the Ethiopian highlands

**DOI:** 10.1371/journal.pone.0341622

**Published:** 2026-02-25

**Authors:** Tilashwork Chanie Alemie, Asmare Wubet, Misganaw Fentahun, Mulugeta Worku, Abreham Awoke, Zime Ambaw, Tadele Amare, Assefa Derebe Zegeye

**Affiliations:** 1 Soil and Water Research Directorate, Adet Agricultural Research Centre, Amhara Regional Agricultural Research Institute, Bahir Dar, Ethiopia; 2 Integrated Watershed Management, Kunzila Integrated Landscape Management and WASH Project, Bahir Dar, Ethiopia; ICAR National Bureau of Soil Survey & Land Use Planning, INDIA

## Abstract

Conventional tillage (CT), which is characterised by intensive soil disturbance and complete residue removal, remains the main farming system in the Ethiopian highlands. However, CT, exacerbated by climate change, has accelerated soil degradation and reduced crop productivity. Conservation agriculture (CA) is promoted as a suitable alternative to counteract these challenges, although evidence of its effectiveness remains limited in the study area. This study evaluated the impacts of CA on runoff, soil loss, infiltration rate, and crop yield by comparing four treatments such as no-tillage + mulching + intercropping (NT + M + In), no-tillage + mulching + crop rotation (NT + M + R), conventional tillage + mulching + crop rotation (CT + M + R), and conventional tillage (CT). Each treatment was implemented at a 10X10 m^2^ plot, while years were considered as replications. Analysis of variance (ANOVA) at a 95% confidence level was applied using R software to evaluate the effects of treatments. The results confirmed significant differences among treatments in reducing runoff and soil loss. Compared to CT, CA practices, NT + M + R, NT + M + In, and CT + M + R reduced runoff by 71, 60, and 45% and soil loss by 88, 82, and 60%, respectively. These treatments also improved cumulative infiltration by 137, 161 and 43%, respectively. These practices also increased maize grain yield by 48, 46, and 47% and faba bean yield by 122, 321, and 91%, respectively. Overall, no-tillage combined with mulching maximised the soil and water conservation benefits of CA, thereby enhancing crop production. Therefore, CA practices, primarily, NT + M + R followed by NT + M + In, and CT + M + R are recommended for wider adoption to improve soil and water conservation, and maize and faba bean production in the study area and similar agro-ecological regions of the world.

## 1. Introduction

The global growth of human population causes the world’s agricultural systems need to produce more food through intensified farming, including Ethiopia, where agriculture is the basis of its economy and the main employment sector [1–3]. The sector is dominated by smallholder farming. In the Ethiopian highlands, which host approximately 80% of the national population, about 85% of residents rely on subsistence livelihoods primarily dependent on traditional rain-fed agriculture [4]. In these highlands and the Amhara region, land/soil degradation has been an ongoing process and caused the population to be food insecure because of the rapid population growth rate and high dependence on inefficient land management practices of conventional tillage, CT [3,5].

CT, which includes postharvest removal of crop residues (for fodder and energy) and intensive soil disturbances using continuous and frequent tillage, causes soil organic matter depletion, soil structure breakdown, delayed hydraulic conductivity that leads to surface flow and increased erosion, and loss of important soil physicochemical properties [6]. This agricultural practice, such as CT, combined with climate change, leads to a decline in the quality of basic natural resources, particularly soil and water, which eventually leads to loss of crop productivity and environmental risk, such as on-site and off-site effects on land and water sources [7,8].

Therefore, considering the relationship between natural resources and community lifestyle, appropriate land (soil) management is an urgent requirement to increase the present crop production at least for food self-sufficiency without altering its potential for future generations [9]. Conservation agriculture (CA) has been initiated as a solution to the problems associated with CT to reduce erosion, enhance soil resilience, and improve crop productivity through minimal soil disturbance, permanent soil cover, and crop rotation [3,10,11].

Intercropping is one of the key practices for agricultural sustainability. By increasing crop diversity, it strengthens agroecosystem functions while decreasing chemical inputs and minimising negative environmental impacts of crop production [12]. Researchers stated that intercropping, which is one of the important sustainable cropping patterns, can improve land use efficiency [13]. The effects of legume-cereal (e.g., soybean-maize) intercropping systems increase the agroecosystem’s productivity with a land equivalent ratio > 1 [14]. Crop rotation is also one of the crucial strategies in sustainable agriculture, providing multiple benefits to both the farming community and the environment [15]. Researchers stated that numerous studies have recommended CA as a soil management approach to increase crop yields, improve soil quality, and reduce soil erosion, land degradation and production costs such as labour and inputs [3]. Similarly, other researchers argue that CA can improve soil quality and crop yield through reducing runoff and soil erosion because it comprises key elements such as reduced tillage, permanent soil cover (mulching) and crop rotations; consequently, it optimises food supply [16,17].

Generally, CA comprises a package of crop production technologies that can achieve sustainable agriculture production and improve smallholder farmers’ livelihood [17]. The mulching component of CA provides a protective blanket of leaves, stems and stalks to the soil. Consequently, it enhances soil productivity by improving its physicochemical properties through soil and water conservation and increasing soil organic matter (OM), the population of micro-organisms (which take over the function of traditional tillage, such as loosening and mixing of the soil), and humus formation [18–21].

Globally, CA is a common practice, but not in tropical Africa, including Ethiopia, the Amhara region and the Yilmana Densa district, where the study area is located [22]. Particularly in the study region, the application of CA is rare since little is known about the comparative benefits of CA practices over CT. Therefore, it is very crucial to test CA practices for their effectiveness on hydro-sedimentology and crop yield in representative areas of rainfall induced severely degraded and intensively cultivated Ethiopian highlands. There is also a suggestion by researchers that the study of CA practice is crucial, especially under conditions of climate change [23]. The representative case study of this research is the Adet agricultural research centre, a research station in Yilmana Densa district of the Amhara region, which is located in the Ethiopian highlands, where soil losses and challenges to food security and environmental sustainability are major characteristics [24,25].

In this study, we hypothesised that CA, in contrast to CT, helps to (1) reduce runoff and soil loss, (2) improve infiltration rate, and (3) increase annual crop production. Therefore, the objective of this study was to examine and compare runoff, soil loss, infiltration rate and major crops yield under CA and CT soil management practices. To attain the objective field experiment was executed, focusing on a representative case study. Then, the effectiveness of CA over CT was evaluated with the R program, using the observed runoff, sediment, and amize and faba bean grain yield data. Furthermore, the impact of CA on soil loss, runoff and grain yield was verified using infiltration rate data, which were also collected at the four experimental plots.

## 2. Materials and methods

### 2.1. Description of the study area

The experiment was conducted using a simple plot, which is a single, uniform experimental unit in which one treatment is applied. The plots (100 m^2^ each) were established at the experimental station of Adet agricultural research centre in northwestern Ethiopia. The experimental site, which has Nitosols soil type, is located nearly 450 km away from Addis Ababa (the capital city of Ethiopia) and 42 km from the capital city of Amhara regional state (Bahir Dar). Geographically, it is located at 11°16’N latitude, 37°29’E longitude, and 2240 meters above sea level [26]. The rainfall, temperature, and relative humidity during the experimental period are summarised in Table 1.

**Table 1:** The rainfall (mm), temperature in °C (maximum: Tmax, minimum: Tmin) and relative humidity (RH in %) of the study area.

Year	Jan	Feb	Mar	Apr	May	Jun	Jul	Aug	Sept	Oct	Nov	Dec	Annual
Rainfall (mm)												
2016	3.18	4.41	47.93	43.34	260.86	218.44	552.06	479.11	188.73	47.53	2.83	0.25	1848.67
2017	0.11	41.82	38.01	134.42	217.13	159.26	507.14	414.08	245.55	37.11	10.84	0.43	1805.9
2018	3.1	37.33	8.03	66.58	71.12	181.07	389.6	343.46	125.5	91.06	88.19	7.4	1412.44
2019	0.02	13.18	43.31	85.14	41.66	164.33	205.52	387.56	317.38	109.28	69	22.79	1459.17
2020	3.13	13.59	10.38	67.13	185.38	220.53	467.2	518.28	392.38	59.56	6.57	1.66	1945.79
2021	39.86	16.8	8.72	95.9	269.52	128.48	769.38	497.63	289.12	61.35	44.68	37.37	2258.81
2022	30.95	8.26	25.28	120.54	80.16	286.23	641.9	314.41	179.34	65.41	5.1	16.21	1773.79
RH (%)													
2016	59.04	46.38	44.79	43.04	70.36	73.83	87.32	87.34	77.97	68.08	55.61	50.02	*63.75*
2017	34.48	54.03	46.32	50.25	67.7	71.41	83.88	87.69	79.62	71.23	64.68	50.64	*63.54*
2018	50.11	45.33	37.08	46.09	53.66	73.98	85.07	86.53	74.37	71.75	67.29	63.1	62.98
2019	42.42	44.82	45.76	49.18	49.92	72.47	80.95	84.77	83.14	72.6	75.03	66.3	64.04
2020	56.21	50.97	36.46	48.38	60.58	75.25	85.72	87.39	78.85	68.47	62.33	59.47	64.22
2021	53.36	48.93	37.05	48.61	66.61	67.73	87.96	86.8	79.26	71.51	67.81	58.94	64.66
2022	63.08	53.19	42.86	48.14	52.43	74.34	88.45	88.68	81.32	73.27	63.9	62.17	66.08
Tmax (°C)													
2016	27.5	32.02	33.03	32.22	28.41	28.33	23.38	22.9	24.33	24.06	26.23	27.34	33.03
2017	29.39	29.37	31.32	31.98	28.87	28.18	24.1	23.82	24.5	24.4	25.79	26.54	31.98
2018	28.54	30.81	31.64	30.49	32.18	27.53	25.17	23.08	24.11	24.31	24.9	26.26	32.18
2019	29.52	30.7	31.06	32.15	32.87	29.41	25.2	24.42	23.78	24.55	24.88	26.29	32.87
2020	28.08	31.27	32.91	32.96	31.39	28.18	24.97	23.49	23.75	23.77	25.08	26.63	32.96
2021	28.15	29.29	32.28	32	29.18	29.42	23.5	23.66	24.17	24.21	24.92	26.89	32.28
2022	26.87	29.26	32.41	32.66	31.47	29.84	22.71	23.17	24.4	24.4	25.07	25.61	32.66
Tmin (°C)													
2016	10.44	12.07	13.65	14.97	14.64	13.6	12.64	12.1	13.2	9.71	6.22	6.09	6.09
2017	5.89	11.78	12.71	13.69	14.92	14.51	13.35	12.36	13.55	10.51	8.84	7.09	5.89
2018	8.58	10.9	10.24	13.62	14.91	13.66	12.43	12.37	11.66	9.68	7.75	10.27	7.75
2019	6.53	10.21	12.42	14.16	15.74	14.69	13.06	13	13.52	10.44	10.88	8.55	6.53
2020	9	11.9	12.8	13.34	13.93	14.28	13.14	11.59	13.78	9.79	7.74	9.46	7.74
2021	9.23	9.83	11.81	12.15	12.8	14.27	12.09	12.57	12.56	9.54	9.99	7.14	7.14
2022	8.76	10.96	13.7	14.99	14.75	14.23	11.96	12.21	12.96	9.74	9.38	9.29	8.76

### 2.2. Treatments, experimental design, and data collection and analysis

The study was conducted during the 2016–2022 cropping seasons at the Adet agricultural research centre in Yilmana Densa district of Amhara region, a soil erosion-prone area of the Ethiopian highlands. For this experiment, four treatments were set. The area follows a single annual cropping cycle (May–October), with farmers typically using a two-year rotation (maize–tef; less commonly maize–faba bean). Although crop rotation was not a treatment factor, maize and faba bean were included in rotation and intercropping, considering the benefits of disrupting pest and disease cycles, and the N-fixation capacity of faba bean. A 30% soil cover at seeding was maintained through the use of precursor crop residue.

In this research, the four treatments were arranged for implementation by considering the three key principles of CA [3]. T1: no-tillage (NT) combined with 30% stubble retention or mulching (M) and intercropping (In), T2: NT combined with M and rotation (R), T3: conventional tillage (CT) combined with M and R, and T4: farmers’ practice or conventional tillage (CT) as described in Table 2. The corrugated iron sheet and roof were used for plots’ hydrological isolation and preventing direct rainfall into runoff-sediment collecting tanks, respectively. During this experiment, the management of land only in CA practices included no tillage, leaving crop stubble in the field and zero grazing. Other agronomic practices and fertilisations were the same for all plots, including CT (T4). Maize (*Zea mays*) and faba bean (*Vicia faba* L.) were grown under rotation and intercropping systems. Both crops are kharif crops sown during the monsoon rainy season, after which the plots were left fallow during the summer period. In the region, the recommended fertiliser application rates are 184N and 92 P_2_O_5_ kg ha ⁻ ¹ for maize, and 18N and 46 P_2_O_5_ kg ha ⁻ ¹ for faba bean.

**Table 2:** Description of treatments and experimental design.

Treatment	Description
T1: NT + M + In	No tillage + Mulching (30%) + Intercropping
T2: NT + M + R	No tillage + Mulching (30%) + Rotation
T3: CT + M + R	Conventional tillage + Mulching (30%) + Rotation
T4: CT	Farmers’ practice (conventional tillage) is characterised by repeated soil tillage and the removal of crop residues, resulting in bare soil conditions at sowing due to residue use for fuel and livestock feed

*Plot size 10 × 10 m; simple plot design; six years of data used as replicates per treatment for ANOVA (except infiltration rate analysis); T3 differed from T4 by retaining 30% of the precursor crop stand.

**Fig 1:**
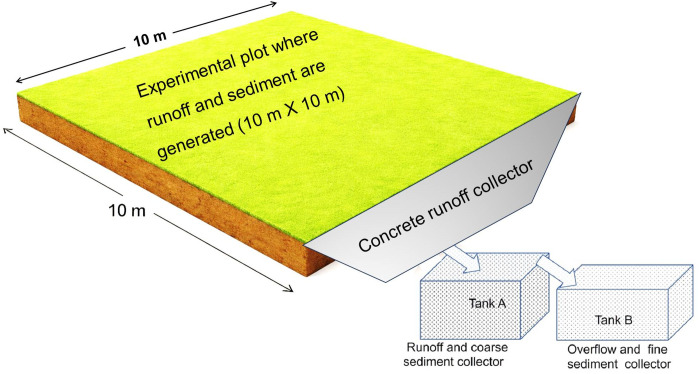
Example of adjacent runoff and sediment collection tanks per plot.

Regarding data collection, runoff and sediment were harvested using two rectangular tanks (set as tank A and B) which were installed at the end of each experimental plot [27], (Fig 1); all these data were recorded in every 24 hours at 8:00 am; regarding sediment data 500 grams and one litre samples were taken from tank A and B respectively per record; next the sediment data were oven-dried [28]. Then, annual runoff and soil loss were calculated based on field records and oven-dried samples. Based on earlier runoff and sediment data, together with field observations, we evaluated the infiltration rate in the sixth year of the experiment to assess its consistency with the observed runoff and sediment trends. To quantify the soil’s capacity to infiltate precipitation, the infiltration rate was measured for each plot using a double-ring infiltrometer during the 6^th^ year of the experiment (2022) [29]. Furthermore, grain yields of maize and faba bean (intercropped and rotated crops during the experiment) were recorded to evaluate the effect of CA on crop yield. Finally, the effects of treatments on runoff, soil loss and crop yield were evaluated, using analysis of variance (ANOVA). Before analysis, the data were checked for consistency and suitability for parametric testing. The ANOVA was conducted using theR program, which allowed comparison of mean values among treatments to determine whether statistically significant differences existed. The treatment effects were considered significant at 95% level of confidence (e.g., *p* < 0.05). But the effects of these practices on infiltration rate were not subjected to inferential statistical tests. Instead, infiltration data were analysed using descriptive statistics and graphical presentations to illustrate trends and relative differences among treatments.

## 3. Results and discussion

**Fig 2:**
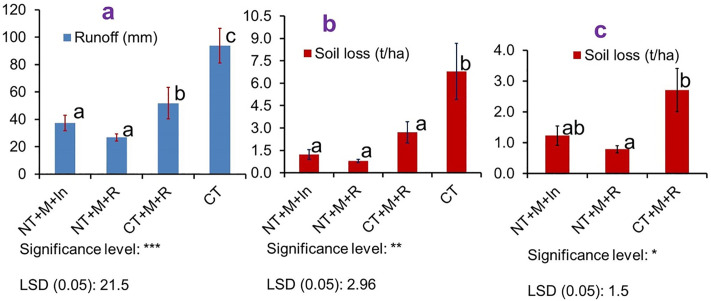
Effect of CA on average annual runoff (a) and soil loss (b and c). b and c show comparisons between four and three treatments, respectively.

In this research, runoff and soil loss revealed responses to treatments, which were the combination of conservation agriculture (CA) and conventional tillage (CT) practices, implemented during six years (2016–2022). It is confirmed that CA practices are effective in reducing runoff and soil loss significantly as compared to farmers’ practice (conventional tillage). The results from the six years of monitoring revealed a highly significant difference among treatments (in the order of T2, T1, T3 and T4) in reducing runoff and soil loss [30]. T2, T1, and T3 lowered runoff by 71, 60, and 45% and soil loss by 88, 82, and 60% as compared to T4 (Fig 2; S1 Table). T2 (NT + M + R) reduces soil loss and runoff more effectively than T1 (NT + M+In). This is because maize–faba bean intercropping in T1 provides less soil cover than sole maize or faba bean cropping in T2, due to competition for space and nutrients and the shading of faba beans by maize. Both T2 and T1, with minimal soil disturbance, perform better in reducing soil loss and runoff than T3 (CT + M + R), which involves greater soil disturbance [31]. Such impact of CA on runoff and soil loss reduction is supported by different studies [9,16,23,32]. This study confirms that no-tillage and mulch (for soil cover) are the key components of conservation agriculture for reducing runoff and soil loss. Researchers reported that the effectiveness of CA is primarily due to no-tillage that leaves the soil undisturbed [21,33]. By keeping the soil undisturbed, no-tillage (NT) minimises the detachment of soil particles and prevents surface sealing and crust formation. Additionally, NT preserves soil organisms and macropores, promotes stronger soil aggregation, and enhances water infiltration. As a result, NT effectively reduces both runoff and soil loss.

Regarding the mulching advantage of CA, a research discovered a clear reduction in runoff and soil losses for the mulch-based practices when compared to CT [34]; the authors stated that the soil loss reduction can be mainly due to the reduction in surface runoff, rain-splash and rill erosion, due to protection by mulch. Compared to bare soil, a 0.50 kg/m^2^ mulch application led to maximum runoff decrease [35]. The importance of maintaining continuous vegetative cover was highlighted, as it leads to the most substantial decreases in both soil erosion and surface runoff [36]. Particularly, crop residue (straw) mulch is affordable and useful in reducing soil loss and runoff over bare soils in regions of the world similar to our study area [37]. Moreover, the long-term results of the study conducted in central Europe showed that the use of mulch is beneficial for farmers by reducing soil losses [38]. In general, mulch reduces runoff and soil loss by protecting the soil from raindrop impact, preventing aggregate breakdown and crust formation, slowing surface flow and promoting higher infiltration, lower erosive forces, and reduced sediment transport.

**Fig 3:**
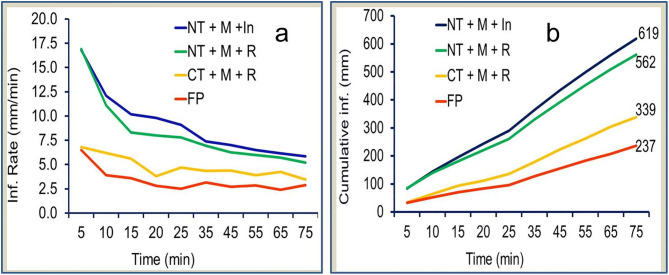
Effect of CA practices on infiltration rate (a) and cumulative infiltration (b).

In this study, CA practices led to lower runoff and soil loss, and enhanced water infiltration rates [39]. As confirmed by the 75-minute infiltration water record, the CA practices in the order of T1, T2, and T3 raised the cumulative infiltration by 161, 137, and 43% compared to T4 (Fig 3 b; S2 Table). This study shows that conservation agriculture (CA) practices foster a stable, porous, and biologically active soil structure that resists surface sealing and improves water infiltration, compared to conventional tillage, by retaining crop residues and minimising soil disturbance. Similarly, significantly higher water infiltration was found on CA fields compared to conventionally ploughed fields in Zambia and Zimbabwe across a wide range of soils [40].

In this experiment, conservation agriculture (CA) improved infiltration and reduced runoff and soil loss by 43%, 45%, and 60%, respectively, when mulch was applied alone, and by 137%, 71%, and 88% when mulch was combined with no-tillage (NT). When mulch was combined with NT and intercropping (In), CA improved infiltration and reduced runoff and soil loss by 161%, 60%, and 82%, respectively. This confirms that mulch and no-tillage are the most important CA elements for reducing runoff and soil loss and improving infiltration over CT. Hence, mulching the soil surface with plant residue is an effective method of soil and water conservation because it reduces surface runoff, increases infiltration of water into the soil and retard soil erosion [41]. This is also in line with the findings of other researchers, such as mulching reduces runoff and soil loss by increasing infiltration of rainfall, with an efficient mulch application found to be 0.25–0.50 kg/m^2^ [39]. The benefits of mulching were explained as it reduced soil surface sealing, as evidenced by higher infiltration rates, and it decreased rainfall and runoff energy for particle detachment and transport, as evidenced by reduced soil loss [39].

**Fig 4:**
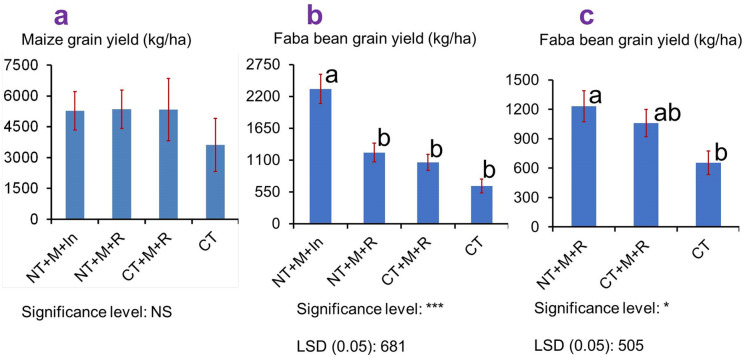
Average grain yield (kg/ha) of maize (a) and faba bean (b,c). b and c are comparisons between four and three treatments, respectively.

Moreover, this research confirms that CA practices maximise maize and faba bean production over CT, although the grain yield data appear inconsistent across years (Table 3). The observed year-to-year variability in maize and faba bean yields is likely driven by climatic fluctuations characteristic of the Ethiopian highlands, particularly variability in rainfall onset, amount, and intra-seasonal distribution. Such rainfall variability strongly influences crop establishment, nutrient availability, and grain filling. In addition, differences in biotic pressures (e.g., pest incidence) and non-optimised crop management practices may have further contributed to yield inconsistencies among years [42]. Considering the standard deviation and average yield, CA practices (T1 and T2) increased the grain yield of maize and faba bean consistently relative to T3 and T4 (Table 3; Fig 4). Based on three years of data, conservation agriculture (CA) practices increased the average maize grain yield by 46–48% compared to farmers’ conventional practices, although this difference was not statistically significant (Fig 4a). Consistent with its effects on reducing runoff and soil loss and raising infiltration rate, conservation agriculture (CA) significantly increased faba bean grain yield compared to conventional tillage (CT). Over three years of data, faba bean grain yield increased under CA, with gains ranging from 62% to 88% compared with CT (Fig 4 b, c). In the intercropping system, CA combined with maize–faba bean intercropping produced a higher faba bean–equivalent yield than the other management practices. This improvement is partly due to the adjusted yield calculation, where the actual faba bean yield is supplemented by the maize yield converted into its faba bean-equivalent value, thereby capturing the full productivity advantage of the intercropped system [43]. When considering sole faba bean production, CA also resulted in significantly higher grain yield relative to CT. This increase can be primarily due to the benefits of mulching (Fig 4c), because mulching (one of the key components of CA) can enhance soil moisture conservation, moderate soil temperature, and improve soil structure [44]. These benefits collectively create more favourable growing conditions, leading to greater faba bean productivity under CA than CT.

**Table 3:** Effect of CA on annual grain yield of maize and faba bean.

Treatments	A: Maize grain yield (kg/ha) per year				
	2017	2019	2021	Average	STDEV
T1	5256	6900	3657	5271	1621
T2	4227	7201	4606	5345	1619
T3	4328	8305	3371	5335	2616
T4	2435	6201	2231	3622	2235
Treatments	B: Faba bean grain yield (kg/ha) per year				
	2018	2020	2022	Average	STDEV
T1	2837	2099	2057	2331	438
T2	921	1452	1322	1232	277
T3	1166	1230	785	1060	241
T4	796	414	452	554	209

In line with the findings of this research, researchers stated that in rainfed farming systems, crop yield potential with conservation agriculture is often greater than the crop yield potential with conventional tillage [45]. The maize–legume systems under conservation agriculture were reported as the best soil management practices to improve grain yield [46]. Similar to the result of this research (i.e., about 1723 kg.ha^-1^ maize yield advantage of CA over CT), a higher yield advantage of maize was produced with CA systems over conventional maize production in southern Africa, where conservation agriculture practices have been promoted mainly for maize-based farming systems [17,47].

## 4. Conclusions and recommendation

This research provides experiment-based evidence that conservation agriculture (CA) substantially reduces runoff and soil loss while improving infiltration and maize and faba bean productivity in the Ethiopian highlands. Among the evaluated CA practices, T2 (NT + M + R) was the most effective, followed by T1 (NT + M+In) and T3 (CT + M + R). Component-level analysis of this study confirmed that mulching and no-tillage are the dominant drivers of hydrological and erosion control benefits. Relative to CT without mulch (T4), mulching alone (M) in T3 reduced runoff, sediment loss, and increased infiltration by 45%, 60%, and 43%, respectively. The addition of no-tillage (NT) further enhanced these benefits by 27%, 28%, and 94%. The maize and faba bean grain yield responses reflected the hydrological and sediment outcomes. T1 and T2 achieved consistently higher maize and faba bean yields than T3 and T4, while T3 led to a higher yield advantage over T4.

Overall, the results identify mulching and no-tillage as the basic elements of effective CA systems and rank T2, T1, and T3 as effective practices for the study area and related agro-ecological regions. These findings recommend a promotion of residue-based no-tillage systems as a priority soil and water conservation strategy for smallholder maize-faba bean production systems in soil erosion-prone highland environments, with direct relevance for food security and climate-resilient agriculture in Ethiopia.

Despite the novelty of its research approach and results, the study was conducted at a single representative site due to resource constraints, which limit broader generalisation. Hence, future research should expand to multi-site trials across the Ethiopian highlands. Besides we suggest the assessment of long-term impacts of CA on soil physico-chemical properties to strengthen evidence-based scaling and policy integration.

## Supporting information

S1 Table Dataset used for runoff and soil loss analysis. (DOCX)

S2 Table Dataset used for treatments comparison based on infiltration rate and cumulative infiltration analysis. (DOCX)
