# Supplementary material for: Conservation agriculture enhances soil and water conservation and crop yield in the Ethiopian highlands
Source: PLoS One. 2026 Feb 25;21(2):e0341622. doi: 10.1371/journal.pone.0341622 (PMC12935193; doi:10.1371/journal.pone.0341622)
Supplement: S1 Table — (DOCX) [file pone.0341622.s001.docx]

S1 Table. Dataset used for runoff and soil loss analysis

| Treatment description | Years per data collection or cropping season | Runoff (mm) | Soil loss (t/ha) |
| --- | --- | --- | --- |
| NT+M+In | 2016-2017 | 17 | 1.24 |
| NT+M+R | 2016-2017 | 20 | 0.99 |
| CT+M+R | 2016-2017 | 29 | 2.58 |
| CT | 2016-2017 | 35 | 2.85 |
| NT+M+In | 2017-2018 | 44 | 0.82 |
| NT+M+R | 2017-2018 | 37 | 0.92 |
| CT+M+R | 2017-2018 | 60 | 1.79 |
| CT | 2017-2018 | 74 | 2.18 |
| NT+M+In | 2018-2019 | 42 | 0.99 |
| NT+M+R | 2018-2019 | 25 | 0.36 |
| CT+M+R | 2018-2019 | 60 | 2.93 |
| CT | 2018-2019 | 105 | 4.36 |
| NT+M+In | 2019-2020 | 43 | 2.96 |
| NT+M+R | 2019-2020 | 35 | 1.24 |
| CT+M+R | 2019-2020 | 113 | 6.32 |
| CT | 2019-2020 | 127 | 8.33 |
| NT+M+In | 2020-2021 | 60 | 1.48 |
| NT+M+R | 2020-2021 | 20 | 0.88 |
| CT+M+R | 2020-2021 | 35 | 1.31 |
| CT | 2020-2021 | 126 | 3.01 |
| NT+M+In | 2021-2022 | 19 | 0.42 |
| NT+M+R | 2021-2022 | 24 | 0.45 |
| CT+M+R | 2021-2022 | 23 | 0.62 |
| CT | 2021-2022 | 77 | 12.18 |
