# Supplementary material for: Conservation agriculture enhances soil and water conservation and crop yield in the Ethiopian highlands
Source: PLoS One. 2026 Feb 25;21(2):e0341622. doi: 10.1371/journal.pone.0341622 (PMC12935193; doi:10.1371/journal.pone.0341622)
Supplement: S2 Table — (DOCX) [file pone.0341622.s002.docx]

S2 Table. Dataset used for treatments comparison based on infiltration rate and cumulative infiltration analysis

| Infiltration rate (mm/min) | | | | | Cumulative infiltration (mm) | | | |
| --- | --- | --- | --- | --- | --- | --- | --- | --- |
| Time (minute) | NT + M + In | NT + M + R | CT + M + R | CT | NT + M + In | NT + M + R | CT + M + R | FP |
| 5 | 16.8 | 16.9 | 6.8 | 6.5 | 84 | 85 | 34 | 33 |
| 10 | 12.1 | 11.1 | 6.2 | 3.9 | 144.5 | 140 | 65 | 52 |
| 15 | 10.2 | 8.3 | 5.6 | 3.6 | 195.5 | 182 | 93 | 70 |
| 20 | 9.8 | 8 | 3.8 | 2.8 | 244.5 | 222 | 112 | 84 |
| 25 | 9.1 | 7.8 | 4.7 | 2.5 | 290 | 261 | 135.5 | 97 |
| 35 | 7.4 | 7 | 4.4 | 3.2 | 364 | 330 | 179 | 128 |
| 45 | 7 | 6.3 | 4.4 | 2.7 | 434 | 393 | 223 | 155 |
| 55 | 6.5 | 6 | 3.9 | 2.9 | 499 | 453 | 262 | 184 |
| 65 | 6.2 | 5.7 | 4.3 | 2.4 | 560.5 | 510 | 304.5 | 208 |
| 75 | 5.9 | 5.2 | 3.5 | 2.9 | 619 | 562 | 339 | 237 |
